# Medical-Legal Partnership as Value-Based Primary Care: Interprofessional Teamwork for Health-Related Social Needs

**DOI:** 10.1007/s11606-026-10402-w

**Published:** 2026-04-20

**Authors:** Jin K. Park, Andrew F. Beck, Keegan Warren, William M. Sage

**Affiliations:** 1 Yale Law School, New Haven, CT USA; 2 Petrie-Flom Center at Harvard Law School, Cambridge, MA USA; 3 University of Cincinnati College of Medicine, Cincinnati, OH USA; 4 Cincinnati Children’s, Cincinnati, OH USA; 5 Texas A&M Health Science Center, Fort Worth, TX USA; 6 Texas A&M University School of Law and College of Medicine, Fort Worth, TX USA

## Abstract

Today’s primary care physicians are being asked to address patients’ health-related social needs (HRSN) as part of their routine care practices. An aging population, with a growing complement of intersecting medical and social needs, increases this burden on primary care. Medical-legal partnerships (MLP) are interprofessional collaborations that combine clinical and legal expertise to address HRSN involving housing, public benefits, employment, and other domains remediable by civil legal assistance. A value-based approach to integrating MLP into the health system may help primary care practices more effectively address HRSN and, in turn, better balance revenue and expenses. At the practice and healthcare system levels, a key operational component is data coordination between medical and legal partners, which will enable MLP to define patient populations whose needs are amenable to legal intervention. At the state and federal levels, policy coordination will play a similar role, assessing existing authorities under Medicaid and other federal programs and advocating for data-justified enhancements. Health systems, hospitals, clinics, and primary care practices should recognize the potential for MLP to improve the value of clinical care delivery for patients with HRSN, their families, and communities.

**Graphical Abstract:**

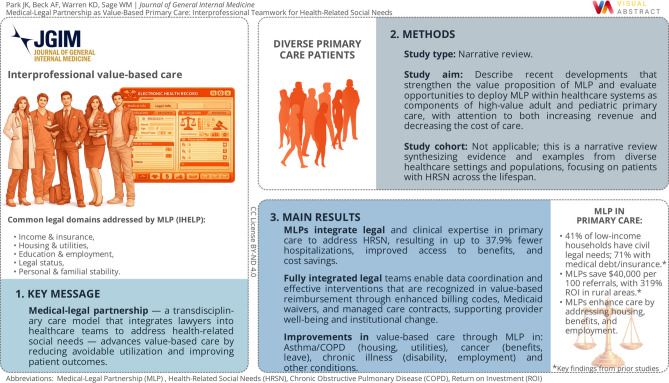

Today’s primary care physicians are being asked to address health-related social needs (HRSN) experienced by diverse patient groups as part of their routine care practices. An aging population, with a growing complement of intersecting medical and non-medical needs, increases this burden on primary care.^1^

HRSN interventions, such as food-as-medicine initiatives, transportation vouchers, and housing assistance programs, are increasingly based on “value”—quality divided by cost, with attention to equity.^2^ This year, for example, the Centers for Medicare & Medicaid Services (CMS) will pilot a value-based primary care model for rural and underserved populations in eight states.^3^

A value-based approach to integrating medical-legal partnership (MLP) into the health system may help primary care practices more effectively address HRSN and, in turn, better balance revenue and expenses. This potential is enhanced given the waning of independent practice; far more primary care physicians are affiliated with medical networks, multispecialty clinics, hospital systems, or academic medical centers now than in previous decades.^4^

Often housed within hospitals or health centers, MLP is a recognized model of interprofessional care delivery.^5^ MLP is distinct from a unidirectional referral network, with significant literature noting the benefits of integration, including improved identification and resolution of health-harming legal needs, enhanced care coordination and patient outcomes, improved provider communication with patients, bidirectional communication between clinicians and lawyers, and higher rates of legal case closure with tangible health impacts such as reduced hospitalizations and restored benefits.^6–8^

MLP is found in all healthcare settings and serves the lifespan. MLP collaborations also enhance the competence of medical teams, enabling them to more effectively address identified needs through training programs on screening, service delivery, and advocacy. There is significant heterogeneity among MLP sites because, as with medical care, legal interventions are aligned with patient needs, but all may be expected to provide a continuum of services, typically including direct legal care to address patients’ HRSN related to housing, public benefits, immigration, education, employment, and other health-harming legal needs.

In this narrative review, we describe recent developments that strengthen the value proposition of MLP, and we evaluate opportunities to deploy MLP within healthcare systems as components of high-value adult and pediatric primary care, with attention paid to both increasing revenue and decreasing the cost of care. We make an important definitional note: there is a conceptual distinction between “HRSN” (individual) and “social determinants of health” (SDOH) (population) that is reflected in value-based contracting and health insurance provisions. Accordingly, in this review we use “HRSN” as a generic term and pivot to “SDOH” only where the applicable payer provision does so.

## THE VALUE PROPOSITION OF MLP—A COMING OF AGE

Since the first modern MLP was created at Boston Medical Center more than 30 years ago, the movement has grown to include hundreds of partnerships nationwide, generally funded through ad hoc grants and philanthropy. That the MLP model thrives without dedicated reimbursement or another stable funding source is testament to its utility and resiliency. In 2014, the Health Resources & Services Administration (HRSA) formally recognized legal aid as an “enabling service,” building momentum to more thoroughly integrate MLP into healthcare delivery and financing, expand system capacity and improve patient outcomes.

A core goal of the value-based care movement is to reorient clinicians from delivering isolated, high-volume services to achieving optimal patient-centered outcomes. Addressing HRSN through legal interventions can amplify the cost-effectiveness of clinical care in codable, measurable ways.^9^ Patients whose social needs are acute or complex—who also tend to generate high healthcare costs—may be most likely to benefit from legal partners as members of the care team.^8^

Among low-income populations, chronic health conditions significantly overlap with unmet civil legal needs. A 2017 Legal Services Corporation report found health problems to be the most common civil legal need, affecting 41% of low-income households nationally.^10^ If one includes healthcare-related issues such as medical debt and insurance, civil legal needs impact 71% of this population, with those affected averaging 2–3 such problems. A 2025 review of legal needs assessments spanning the last 30 years found that, across the socioeconomic spectrum, the most frequently reported consequences of legal needs are harm to health, financial instability, and stress.^11^ That review also found that people who report health impacts from legal issues visit healthcare providers more often. Thus, MLP represents a critical opportunity to address a root cause of avoidable healthcare utilization and to disrupt health-harming cycles by intervening at the point of care.

Specifying gaps in care that are amenable to MLP and the costs and benefits of filling them with MLP capabilities is a priority.^12^ Programmatic costs are typically covered through blended and braided funding. Though budgets vary, a 2019 nationwide survey found that the median MLP budget for health centers was $75,000, with a median contribution of only $28,000 by the health center.^13^ Nonetheless, fiscal benefits primarily accrue to health systems via lower utilization or increased coverage of care. Patient-client access to financially stabilizing benefits and avoidance of medical debt is also enhanced by MLP.^14^

One recent study found that referral to an MLP reduced hospitalization rates experienced by low-income children by 37.9%, or approximately three fewer hospitalizations each year per 100 pediatric patients, which the authors estimated as generating annual healthcare savings of roughly $40,000.^15,16^ Another empirical study demonstrated a 319% return on investment over 3 years for MLP legal care delivered in a rural setting.^17^ No empirical study to date directly quantifies woodwork effects in the context of MLP, likely because woodwork analyses traditionally focus on entitlement programs where outreach can drive enrollment surges. In contrast, a lawyer’s “panel” is constrained by responsible caseload management, such that mitigation occurs through targeted triage of high-need cases—aligning with value-based contracting principles.

## A CASE STUDY

To illustrate how MLP functions as value-based primary care, we focus on a representative case rather than a catalog of possible interventions. The case is intended to be clinically recognizable, to trace a clear pathway from health-related social need to legal action to health and cost outcomes, and to demonstrate concretely how medical and legal teams collaborate in real time. The domain of health-related legal interventions is extraordinarily broad—mirroring the breadth of law itself—so no single article can comprehensively enumerate all possible health conditions, legal claims, and procedural postures; instead, this case anchors the discussion while the surrounding text and Table 1 generalize to wider populations and financing models.

C. is a 31-year-old man without health insurance who recently presented to the emergency department (ED) for the third time this year with an asthma exacerbation. The severity of this presentation results in hospitalization. Irritants in the substandard housing where he lives triggered his asthma, but the landlord has ignored concerns voiced by him and his spouse. He has no regular source of care and does not consistently receive maintenance or rescue medications. After hospital discharge, he schedules a follow-up appointment with a primary care physician at a nearby clinic. In the waiting room, he completes a social needs screening tool offered to him by a medical assistant in which he checks a box about housing concerns. At his visit, the physician prescribes a biologic interleukin inhibitor, which he cannot afford without insurance. He should be eligible for Medicaid, but his efforts to enroll have yet to yield a response from the state program. As a result of C.’s positive social needs screen and his difficulties with Medicaid enrollment, the physician refers C. to the clinic’s MLP legal team.

**Table 1:** Sample MLP Value Measures

	Sample measures			
Quality	Structural *Capacity, systems, and processes* ◦ Number of FTE legal professionals ◦ Ratio of patients to legal team members ◦ In primary care settings, the ratio of legal professionals to physicians and to non-physician providers (NPs, PAs, RNs, CNS, etc.) ◦ Amount of protected clinician time for MLP championship ◦ Amount of protected administrative time for MLP championship ◦ Amount of MLP legal team time dedicated to institutional change (e.g., improving healthcare partner’s policies or procedures)	Process *Actions to improve health* ◦ Number of patients screened for health-harming legal needs ◦ Number of referrals to/from legal team ◦ Number and type of cases opened ◦ Number and type of cases closed ◦ Number of persons helped ◦ Legal time spent per case type and intervention type ◦ Number and distribution of legal service types deployed (e.g., counsel and advice, limited action, negotiated settlement, administrative hearing, judicial hearing) ◦ Percentage of referrals resulting in a legal intervention ◦ Number of curbside consults regarding legal needs ◦ Number of patient and provider trainings ◦ Financial gain from visits upcoded due to enhanced evidence and impact of patient social needs on medical decision-making ◦ Number and value of reimbursed team-based encounters ◦ Number of new technological processes incorporating MLP ◦ Number of institutional changes (e.g., attorney involvement in complex case conferences or group medical visits)		Outcomes *Impact on health* ◦ Legal outcomes for patients ◦ Patient satisfaction with legal services ◦ Benefits obtained and financial liability avoided for patient ◦ Financial outcomes for healthcare entity ◦ Patient-reported health outcomes (e.g., sense of stress, symptom-free days) ◦ Provider-reported outcomes (e.g., relief of moral distress, efficacy) ◦ Care completion ◦ Decrease in missed appointments ◦ Completion of preventive care visits ◦ Completion of USPSTF-recommended screening interventions ◦ Core HEDIS measures ◦ Acute care reductions ◦ ED visits ◦ Inpatient hospitalizations ◦ ICU admissions ◦ Avoidable outpatient encounters ◦ Patients retained/not lost to care due to removal of access barriers
Cost	Patient-facing costs ◦ Salary and benefits for on-the-ground and supervisory legal personnel ◦ Salary and benefits for clinical, administrative, and other healthcare champions (staff dedicated to ensuring the success and sustainability of the MLP) ◦ Interpreter services ◦ Travel		Back-office costs ◦ Legal technology and support services ◦ Facility overhead for consultations and telecommunications ◦ Billing and program administration	

Asthma exacerbations are common causes of ED visits and hospitalizations, especially but not exclusively for children. Co-existing adult pulmonary conditions such as COPD can precipitate similar events. Much of this morbidity is avoidable, and its burden is distributed unequally along racial, ethnic, socioeconomic, and geographical lines.^18^

MLP services can reduce avoidable asthma-related care by helping primary care settings target unmet housing needs and public benefit denials and delays.^19,20^ A 4-year retrospective of low-income pediatric patients with any MLP intervention was associated with reductions in hospitalizations by 69.7% and total asthma exacerbation-related encounters by 44.2%, underscoring the impact of integrated legal care.^21^ In the case introduced above, the MLP legal team might advocate for healthier housing conditions in line with state law or local ordinance, removing harmful symptom triggers at home. MLP legal care also could assist in successful Medicaid enrollment, helping this individual overcome barriers to receipt of needed medications and preventive care (including representing C. at a fair hearing should medication coverage be denied).

Positive effects could extend to the patient’s family members, including children and frail elderly persons. Clinicians may, too, experience benefits—from knowledge growth to job satisfaction.^22^ Moreover, enforced rights to healthy housing for one family are likely to result in changes for similarly situated families, either by shining a spotlight that creates public pressure for improvements or by enabling population-level legal action by the MLP legal team.^23^

Not only can MLP prevent some individuals from becoming so sick as to require high-acuity, necessarily riskier care, MLP also can enrich and focus medical information in ways that guide and improve preventive clinical management.^24^ For certain indications, MLP therefore may meet the evidentiary standards needed to participate in value-oriented federal demonstration programs such as the Community-based Care Transitions Program (CCTP), which supports community-based organizations that can reduce hospital readmissions for high-risk Medicare beneficiaries.^25^

Robust systems to record quality and cost are important for quantitative assessment of the impact of a given MLP intervention on the value equation for a single practice setting, a provider network, or an entire healthcare system. A structure-process-outcome model can be used to introduce MLP quality measures.^26^ Table 1 adapts Donabedian’s framework with structural metrics assessing capacity (e.g., legal full-time employees, clinician time), process measures tracking interventions (e.g., referrals closed, trainings provided), and outcomes capturing health and financial impacts (e.g., emergency department visit reductions, upcoded visits, benefits secured) alongside costs (e.g., staffing)—enabling practices to quantify value for reimbursement in alignment with field data. Measures like those in Table 1 can quantify both quality (numerator) and cost (denominator) in most if not all primary care settings. Alternative specifications are possible, including resource use, staffing costs (e.g., protected time for MLP champions), patient-reported outcomes, and other metrics specific to a given value-based contract.

## STEPS TO INTEGRATE MLP ACTIVITIES INTO VALUE-BASED HEALTHCARE FINANCING STREAMS

**Figure 1:**
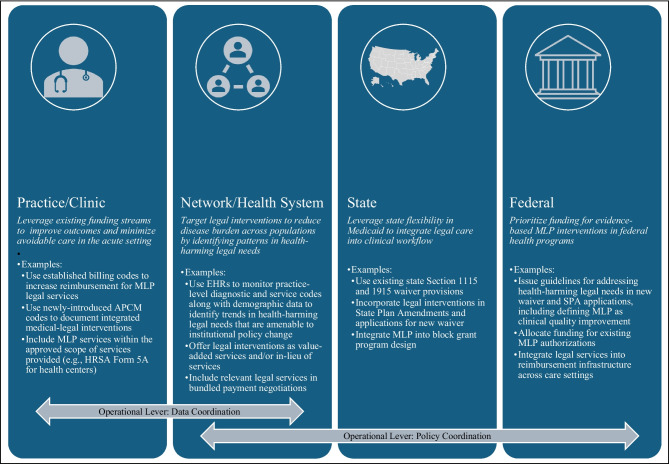
Step-wise value-based approach to integrate medical-legal partnership services into the US health system.

The incorporation of MLP activities into value-based healthcare delivery requires action at five levels across the healthcare system (Fig. 1). Because clinical needs and financial capacity vary, the activities listed can be adapted to setting and circumstance.

### Documenting MLP Legal Care in Connection with Existing Medical Billing Codes

Adequately documenting all MLP services recommended or provided, including legal services, is critical both when direct reimbursement is available^27^ and when applying alternative payment methodologies. For example, new codes for unmet social needs identified by MLP can confer eligibility for enhanced medical reimbursement because those needs complicate medical decision-making in primary care settings and EDs. MLP legal teams also can improve uptake of paid, team-based interventions, such as Medicare’s advance care planning allowance.

When C., the individual with asthma in the example above, presents for care, the MLP medical team would screen for unmet legal needs (e.g., substandard housing conditions, public benefit denial or delay) as part of taking a standardized social history. If unmet social needs complicate medical decision-making, the medical team could upcode its billing to reflect that complexity, and they could refer the patient or family to the MLP legal team for assistance.

In parallel, legal service providers routinely document client names, contact and neighborhood information, and demographic data such as sex, gender, race, and income, maintained in case management software similar to electronic health records. Metrics promulgated by the Legal Services Corporation (LSC) are mandatory for its legal aid providers and are used voluntarily by many non-LSC-funded legal services organizations. Other public interest attorneys use the National Subject Matter Index or the Uniform Task Based Management System. Every MLP therefore can capture the legal diagnosis (e.g., LSC problem code), the acuity of the need (e.g., LSC scope of services code), and social and financial outcomes for clients they serve.

MLP services are collaborative between the medical and legal partners; for example, an internist and an attorney building C.’s case for housing remediation grounded in both medicine (e.g., the impact of environmental exposure on asthma symptoms) and law (e.g., applicable building and housing code violations). Advocacy efforts range from legal advice for the family to correspondence with the landlord to representation in court. At case closing, the MLP legal team would document the interventions provided using established service codes, including time spent, the legal outcome, and any financial benefit accruing to (or financial cost avoided by) the patient-family. This legal documentation may justify medical billing upcoding or facilitate documentation of a health-related social need contemplated by the International Classification of Diseases, 10th Revision (ICD-10) in the Z55–Z65 series.^28^ The MLP medical team would evaluate the care experience and assess the potential impact of the legal outcome on C.’s health, including projected preventive and acute care visits.^29^ Measurable benefit may also accrue to members of the care team by offering them concrete, meaningful options for complex patients.^30^ Over the long term, this can reduce moral injury, burnout, and costly attrition among both clinicians and support staff.^31^

New team-based billing codes offer additional opportunity to realize value from MLP. Medicare’s Strengthen Primary Care initiative, a recently launched effort to move payment away from volume-dependent fee-for-service visits, includes streamlining reimbursement for team-based care.^32^ New “G-codes” for Advanced Primary Care Management “stratify APCM services based on how chronic medical conditions interact with increased risk associated with social determinants of health (SDOH) factors,” enabling comprehensive care management that includes “systematic needs assessment (medical and psychosocial)” as well as “system-based approaches to ensure receipt of preventive services.”

Consistent with current guidance for team-based codes (e.g., Medicare codes 99497 and 99498), both physicians and non-physician clinicians can bill using G-codes for APCM services, which can be provided by auxiliary personnel and therefore. would enable a primary care practice to be reimbursed for the legal services provided to C., the patient with asthma above. Similarly, an annual well visit at which patients and MLP team members discuss both medical and social contributors to ill health would be a reimbursable venue to screen for and address health-harming legal needs.^33^

### Continuing to Expand Medicaid Reimbursement

Detailed, consistent documentation of MLP’s value equation for primary care may support further expansions of Medicaid reimbursement for legal services. Many MLP sites have received Medicaid dollars through one-time grants from a single state or Medicaid insurer. For legal services to be systematically reimbursable by Medicaid, however, a state must obtain permission from CMS.

There are two primary mechanisms for doing so: a state plan amendment (SPA) or the suspension of specific federal requirements through a Section 1115 or Section 1915 waiver request. No state has amended its state plan expressly to include legal services, but several state Medicaid laws have expansive case management or care coordination provisions that could encompass the provision of MLP legal services.

MLPs should also review Medicaid waivers that have been approved in their states of operation for care provision focused on SDOH or HRSN. MLP legal services may fit within one or both waiver definitions, depending on the state.^34^ Twenty-four states currently have SDOH provisions, and twelve have pending requests before CMS.

CMS provided guidance to states wishing to address HRSNs in November 2023, and, as of January 2025, eight states had approved applications under the HRSN framework. On March 4, 2025, however, CMS rescinded its HRSN bulletins, stating that future waiver applications for HRSN services will be reviewed on a case-by-case basis.

Thus far, only North Carolina has obtained express permission for Medicaid reimbursement of legal services at a set rate, which are limited to advice and counsel for certain patients.^35^ This would cover consultations on legal rights to healthy housing for our hypothetical patient C., but it would not include the full complement of integrated medical-legal services that the MLP model makes available.

MLPs advocating for new Medicaid waivers should recognize the need to demonstrate budget neutrality under federal law, meaning that costs with the waiver will not exceed costs without it. This implies rigorous oversight of expenditures, which in turn requires legal services organizations to develop more precise means of cost measurement, including a billing approach that is informationally aligned with that of healthcare providers.

A fair payment rate in the waiver context should include appropriate eligibility criteria for acute legal care, while also promoting integrated legal expertise as part of population health management. At the same time, both states submitting MLP waiver requests and CMS evaluating them should recognize that hierarchies of evidence (e.g., randomized controlled trials (RCT)) and associated data science developed for medical outcomes may not be appropriate for legal interventions.^36^

### Reimbursing MLP Legal Services Through Managed Care Contracts

In 40 states plus Washington, D.C., Medicaid is provided through managed care organizations (MCOs), or health insurers with a Medicaid line of service. MCOs may choose to fund MLP because of its cost-effectiveness, or primary care clinics that contract with them may agree to do so.

Since 2016, Medicaid MCOs have been required to spend a majority of their revenue on clinical services rather than administrative overhead or profit based on a measure called the “medical-loss ratio” (MLR).^37^ To the extent they improve healthcare quality, MLP legal services fit within the definition for “value-added services” (VAS) or “in lieu of services” (ILOS), which place them on the clinical side of the MLR calculation. Tenants’ rights education is within the ILOS HRSN schema, making MLP services doubly attractive to a Medicaid MCO in which our hypothetical asthmatic patient, C., might be enrolled.

MLP legal services also have been deployed successfully to benefit health systems in risk-based contracts with MCOs outside of Medicaid.^38^ For example, an outpatient mental health clinic in Indiana faced penalties whenever its patients occupied more than a certain number of inpatient beds. The MLP attorney secured wrongly denied disability benefits for a medically stabilized patient who had remained institutionalized, enabling the patient to afford housing and be discharged—in turn helping the clinic meet its occupancy goals. In our case study, if legal advocacy resulted in healthier living conditions for C., and that advocacy reduced C.’s healthcare utilization (ED use, admissions, length of stay), a hospital receiving fixed or bundled payments would realize the savings, as might a primary care group receiving value-based financial incentives.

Because legal advocacy has spillover benefits across households and communities, that hospital or primary care group also may realize savings for other patients in its service geography. Indeed, C. may have family members whose health also improves, or C.’s landlord may remediate respiratory triggers in other apartments or properties because of the attorney’s advocacy. For these types of legal services, MLPs can use geographic data to estimate multiplier effects, enhancing the value proposition of individual representations.

Health plans can take this approach as well. For example, were C. a member of AmeriHealth in Washington, D.C., extended legal representation that resulted in healthier housing would be valued at $10,000. Half of that amount would be returned to the MLP legal team as a direct performance-based contractor, while the health plan would realize the other half as savings.^39^ A sponsoring healthcare organization following the MLP model could similarly hold a risk-based contract and receive half the savings.

### Other Value-Based Financing Approaches for MLP

**Figure 2:**
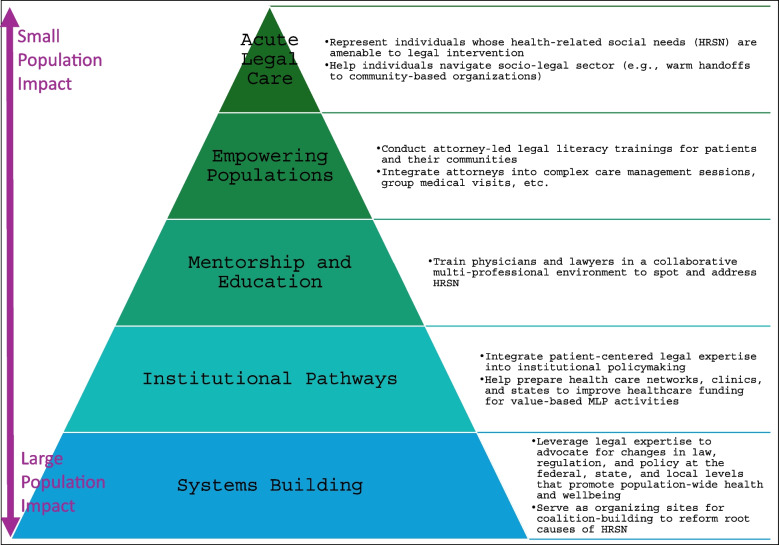
VBC-informed activities performed by MLPs.

The MLP model represents a continuum of legal care (Fig. 2). In addition to individual representation, it supplies training for patients and providers, and it leverages patient-specific legal expertise to inform change at the level of a practice, healthcare system, or community. MLP legal teams may contribute to case conferences, workgroups, group medical visits, outreach efforts, and research activities. MLP are also engaged in building national coalitions to advocate for regulatory and legislative reforms at the local, state, and federal levels.^40^ These services may supplement other sources of financial support for MLP while helping both clinical and legal organizations meet their benchmarks under value-based contracting with other types of funders.

To give one example, a federally qualified health center in Texas negotiated a bundled rate with a county-based medical assistance plan for an integrated pain management program as part of its response to the opioid epidemic. The MLP legal team dedicated an attorney to serving that population, including through legal education trainings, multiprofessional case management sessions, and group medical visits.^41^ Robust sharing of medical and legal information in the health center’s electronic health record made this possible, and the legal team carefully documented health-harming legal needs, interventions, and outcomes to demonstrate its cost-effectiveness.

Block grants administered by the federal Administration for Children and Families are another potential source of funding for civil legal services.^42^ These include the Title V Maternal and Child Health (MCH) Services Block Grant, in which the federal government partners with states and localities. In Ohio and certain other states, the MCH block grant takes a systematic “life course” approach to reducing harms that can be attributed to SDOH.^43^ To that end, Ohio is supporting MLP in four maternity centers using MCH funds.^44^

Similarly, the Substance Abuse and Mental Health Services Administration (SAMHSA) has awarded demonstration grants to 14 states and Washington, D.C., to establish Certified Community Behavioral Health Clinics (CCBHCs) to improve mental health treatment and prevention. The Care Plus New Jersey MLP, including a Community Health Law Project, is a core part of the state’s CCBHC SDOH services.^45^

Cities and counties are also important sources of MLP funding. Since 2002, New York City Health + Hospitals—the largest municipal health system in the USA—has partnered with LegalHealth, a division of the New York Legal Assistance Group. From 2015 through 2020, LegalHealth’s work was funded through the Delivery System Reform Incentive Payment (DSRIP) Program under New York State’s Medicaid 1115 waiver.^46^ Los Angeles County has funded MLP indirectly through its drawdown of state Medicaid funds since 2018, and California is currently working to fund MLP directly as part of the Medi-Cal program.

## CONCLUSION

Health systems, hospitals, clinics, and primary care practices should recognize the potential for MLP to improve the value of clinical care delivery for patients with HRSN, their families, and communities. To do so, MLPs and their clinical partners must measure health outcomes, survey patient experience, and determine actual costs associated with both the medical and legal services that are delivered. Working together, organizations also can embed the MLP model in value-based care contracting with suitable benchmarks and definitions of risk. At the practice and healthcare system levels, a key operational component is data coordination between medical and legal partners, which will enable MLP to define patient populations whose needs are amenable to legal intervention. At the state and federal levels, policy coordination will play a similar role, assessing existing authorities under Medicaid and other federal programs and advocating for data-justified enhancements. Over the longer term, advancing MLP requires rigorous data infrastructure—standardized metrics for health/legal outcomes, interoperable EHR-legal systems, and predictive analytics for HRSN patterns amenable to legal interventions. There is also a likely role for generative artificial intelligence (AI) to enable MLP to most effectively connect patient-level remedies to population health justice, powering both value-based contracts and policy reforms.
